# Season of birth and diagnosis of children with leukaemia: an analysis of over 15 000 UK cases occurring from 1953-95

**DOI:** 10.1054/bjoc.2000.1575

**Published:** 2001-02

**Authors:** C D Higgins, I dos-Santos-Silva, C A Stiller, A J Swerdlow

**Affiliations:** Cancer and Public Health Unit, Department of Epidemiology and Population Health, London School of Hygiene and Tropical Medicine, London, WC1E 7HT, UK; Childhood Cancer Research Group, Department of Paediatrics, University of Oxford, 57 Woodstock Road, Oxford, OX2 6HJ, UK

**Keywords:** cancer, childhood leukaemia, season, birth, diagnosis, infection

## Abstract

If infections are involved in the aetiology of childhood leukaemia then seasonal variation in the birth or onset dates of the malignancy may be apparent. Previous studies that have examined seasonality of these dates have produced conflicting results. Using population-based data from the National Registry of Childhood Tumours we conducted a larger study than any to date of 15 835 cases of childhood leukaemia born and diagnosed in the UK between 1953–95. We found no evidence of seasonality in either month of birth or month of diagnosis overall or in any subgroups by age, sex, histology or immunophenotype. We did however find a significant (*P*= 0.01) February peak in month of birth for cases born before 1960 and a significant (*P*= 0.02) August peak in month of diagnosis for those diagnosed before 1962. Whilst these findings may be due to chance they are also consistent with changes over time in the seasonality of exposure, or immunological response, to a relevant infection. Changes in the seasonal variation in the fatality rate of a pre-leukaemic illness, such as pneumonia, could be another explanation. © 2001 Cancer Research Campaign http://www.bjcancer.com


*British Journal of Cancer* (2001) **84**(3), 406–412
© 2001 Cancer Research Campaign
doi: 10.1054/ bjoc.2000.1575, available online at http://www.idealibrary.com on IDEAL®

### Season of birth and diagnosis of children with leukaemia: an analysis of over 15 000 UK cases occurring from 1953–95

**CD Higgins[1], I dos-Santos-Silva[1], CA Stiller[2] and AJ Swerdlow[1]**

[1]Cancer and Public Health Unit, Department of Epidemiology and Population Health, London School of Hygiene and Tropical Medicine, London WC1E 7HT, UK; [2]Childhood Cancer Research Group, Department of Paediatrics, University of Oxford, 57 Woodstock Road, Oxford OX2 6HJ, UK

**Summary** If infections are involved in the aetiology of childhood leukaemia then seasonal variation in the birth or onset dates of the malignancy may be apparent. Previous studies that have examined seasonality of these dates have produced conflicting results. Using population-based data from the National Registry of Childhood Tumours we conducted a larger study than any to date of 15 835 cases of childhood leukaemia born and diagnosed in the UK between 1953–95. We found no evidence of seasonality in either month of birth or month of diagnosis overall or in any subgroups by age, sex, histology or immunophenotype. We did however find a significant ($P$ = 0.01) February peak in month of birth for cases born before 1960 and a significant ($P$ = 0.02) August peak in month of diagnosis for those diagnosed before 1962. Whilst these findings may be due to chance they are also consistent with changes over time in the seasonality of exposure, or immunological response, to a relevant infection. Changes in the seasonal variation in the fatality rate of a pre-leukaemic illness, such as pneumonia, could be another explanation. © 2001 Cancer Research Campaign http://www.bjcancer.com

**Keywords**: cancer; childhood leukaemia; season; birth; diagnosis; infection

The aetiology of childhood leukaemia remains largely unknown despite considerable research over the past 40 years. One hypothesis put forward is that it may be due to a common infectious agent (Kellett, 1937) and there is a growing body of evidence to support this (Doll, 1999). One way to substantiate this hypothesis is to search for seasonality of birth or diagnosis date, as this might indicate aetiology by an infective agent which itself varies seasonally.

A number of studies have investigated the possibility of seasonal variation in the birth or onset dates of leukaemia cases. While many have not revealed an obvious seasonal pattern (e.g. Douglas et al, 1999), others have found significant seasonal variations (e.g., Badrinath et al, 1997). There are a number of possible reasons why inconsistent results may have occurred. Some studies (e.g. Mainwaring, 1966) have been based on a small number of cases and have lacked statistical power. Most of the studies examined season of diagnosis (e.g. Badrinath et al, 1997) and only a few investigated season of birth (e.g. Meltzer et al, 1996). A seasonal infective agent acting prenatally or soon after birth may only be apparent from examination of month of birth whilst an agent acting during childhood with a short incubation period from infection to clinical disease may be more apparent by examination of onset date. Some studies (e.g. Stark and Mantel, 1967) combined all childhood leukaemias without taking account of histology or age. It is possible that seasonality may be more pronounced within subtypes of leukaemia or within particular age-groups. Animal models have suggested that oncogenic viruses are likely to have their greatest effect on newborns and that a seasonal agent acting prenatally or soon after birth may produce seasonal fluctuations that are only evident among cases of infant leukaemia (Ederer et al, 1965). Finally, a variety of statistical techniques have been used to search for seasonality, from crude methods such as summer: winter ratios to more complex modelling techniques, and these different approaches may be one of the reasons for different findings.

Using data from a population-based registry of childhood cancers we have therefore carried out a larger study than any to date to investigate whether seasonal variation exists in the birth or diagnosis dates of children with leukaemia.

*Received 29 June 2000*
*Revised 9 October 2000*
*Accepted 10 October 2000*

*Correspondence to:* CD Higgins

### MATERIALS AND METHODS

The National Registry of Childhood Tumours (NRCT) contains population-based data on cases of childhood cancer occurring in England, Wales and Scotland since 1953. Data were collected on fatal cases of leukaemia until 1961 (at ages 0–9 years until 1954 and ages 0–14 years thereafter for England and Wales, and ages 0–9 years until 1955 and ages 0–15 years thereafter for Scotland) and incident cases at ages 0–14 from 1962 onwards. Completeness of registration has been high – at least 98% throughout the period of study. Further details relating to the methodology, completeness and accuracy of the registry have been reported elsewhere (Stiller et al, 1995). For the present study, we extracted from the register for each case of leukaemia born and diagnosed between 1953 and 1995, data on the child's sex, date of birth, date of diagnosis and histological type. Before 1962 childhood leukaemia was virtually always fatal and cases for this period were ascertained from death certificates. Dates of diagnosis for these cases were obtained retrospectively from hospital records. If the hospital records could not be located (approx. 15% of the pre-1962 deaths) the date of diagnosis was regarded as 'unknown' and the case was excluded from the present analyses. Cases diagnosed during the study period but born before 1953 were also excluded.

We also extracted information on whether the child had Down's Syndrome (only available for cases diagnosed from 1971 onwards) and on the immunophenotype of the lymphocytic leukaemias (only available for cases diagnosed from 1976 onwards).

For month of birth analyses, sex-specific monthly birth counts for England and Wales from 1953 to 1995 were obtained from publications of the Office for National Statistics (Registrar General's Statistical Review, 1955–75; OPCS Birth Statistics, 1976–97). For Scotland, special tabulations of monthly birth counts were obtained from the General Register Office for Scotland (GRO, personal communication), but sex-specific counts were only available on an annual basis, not for each month separately. To estimate monthly birth counts by sex for Scotland, therefore, we applied the annual sex-ratio to the monthly totals for both sexes combined. This is highly unlikely to have influenced the results of any sex-specific analyses since the proportion of leukaemia cases that were diagnosed in Scotland is small (only 10% of the overall total) and examination of the England and Wales figures showed that seasonal variation in the sex-ratio of births is negligible. The monthly counts for England, Wales and Scotland were then combined and used as denominators to calculate aggregate incidence rates of leukaemia for each month of birth.

For month of diagnosis analyses, delays in leukaemia diagnosis caused by the holiday period over Christmas and New Year will have produced an underestimate of the actual December incidence and a corresponding overestimate of the January incidence. For this reason, the mid-point of each month, rather than the first day, was used to divide the year into 12 periods for the month of diagnosis analyses. The number of cases diagnosed during each of these periods was then divided by the number of days within the period (with the assumption that the population-at-risk remained constant throughout each year) and the degree of seasonality and period of peak occurrence were estimated by the methods described below. Since a cosinor model allows estimation of the exact day when the peak occurs, it was possible to determine whether the peak occurred during the first or second half of the period and therefore the actual calendar month in which it occurred. Cases with an unknown day of diagnosis were randomly allocated to the first or second half of the month. To provide an estimate of how much the results may have been affected by this allocation we also conducted analyses assigning all of them to the first half and then all of them to the second half of the month.

Patterns of seasonality were examined using cosinor (or harmonic) analyses (Edwards, 1961). Full details of this technique can be found in earlier reports that have used this approach to model the seasonality of childhood leukaemia (Harris et al, 1987; Meltzer et al, 1996; Westerbeek et al, 1998; Douglas et al, 1999). The adequacy of the method has been compared to several alternatives and found to be the most appropriate to examine data of this nature (Marrero, 1983). In a more recent article, Machin and Gao (2000) recommended, for comparative reasons, that all seasonality analyses use this approach to obtain an estimate of the strength, as well as the timing, of the peak. In brief, Poisson regression was used to model the rate of leukaemia as a harmonic function of month by fitting a sine curve to the data and obtaining an estimate of the peak month along with its amplitude (percent by which rate at peak month is greater than mean rate for all months combined; for example, an amplitude of 20% corresponds to the rate in the peak month being 20% greater than the mean rate for the whole year). The statistical significance of the peak was tested using the likelihood ratio test, by comparing the goodness of fit of the harmonic model to the goodness of fit of the model assuming a constant rate throughout the year. To take account of the rising incidence rates between 1962 and the mid 1970s and then their relative stability thereafter, a quadratic term for calendar month (Jan 1953 = 1, Feb 1953 = 2, . . . Dec 1995 = 516) of registration or birth was included in each of the harmonic models. A quadratic term was found to provide a better fit than a simple linear term. Models without the temporal trend adjustment were also fitted in order to compare with findings in previous studies. The cosinor analyses were conducted on monthly, rather than weekly or daily, rates of leukaemia because it was felt that this would adequately capture the broad seasonal variation that we were attempting to disclose. Also, a substantial degree of statistical power would have been lost through analyses across such a larger number of strata (52 for a weekly and 365 for a daily analyses). All analyses were carried out using the STATA statistical software (StataCorp, 1997).

Season of birth and season of diagnosis analyses were firstly carried out on the total dataset and then separately according to sex, age (<1 year, 1–4 years, 5–9 years, 10+ years) and histology (lymphocytic leukaemia, all other leukaemias). Infant leukaemias were analysed in two further age groups (<6 months, ≥6 months). For lymphocytic leukaemias diagnosed from 1976 onwards, further analyses were conducted according to immunophenotype (common, B-cell, T-cell, null-cell, unknown) and an alternative age stratification: <1 year, 1 to 6 years (when common and pre-B acute lymphocytic leukaemia (ALL) are most common) and over 6 years. Season of diagnosis analyses were also conducted according to calendar year of registration (pre-1962, when only fatal cases were registered and a date of diagnosis was obtained retrospectively from hospital records; 1962 onwards, when all incident cases were registered) and season of birth analyses according to year of birth (five birth cohorts: <1960, 1960–69, 1970–79, 1980–89, ≥1990). Both month of birth and month of diagnosis analyses were also conducted separately on the small number of children who were recorded as having Down's syndrome.

Seasonal patterns might hypothetically arise as a result of biases in the registration system. Therefore similar seasonality analyses were conducted on the national registry data for childhood cancers other than leukaemia diagnosed between 1962 and 1995 (data on incident cases were not available before 1962).

### RESULTS

A total of 16 040 cases of leukaemia in children born and diagnosed during 1953–1995 were registered. Information on year or month of diagnosis was missing for 205 (1.3%) of the cases, leaving a total of 15 835. Information on day of diagnosis was missing for 2561 (16%). The allocation of all of these cases to the first half and then all of them to the second half of the month made little difference to the results of the seasonality analyses.

Table 1 shows descriptive characteristics of the cases, together with the results of the season of birth analyses before and after adjustment for cohort trends in the incidence of leukaemia. The slight majority of cases were in males (56%), only 6% were diagnosed during infancy and most were lymphocytic (73%). Immunophenotype was known for 5451 (80%) of the 6838

© 2001 Cancer Research Campaign

**Table 1** Descriptive characteristics of cases and seasonality of month of birth

| | | | Harmonic analyses | | | | | |
|---|---|---|---|---|---|---|---|---|
| | | | Unadjusted | | | Adjusted[a] | | |
| | No. | % | Peak month | Amplitude[b] (%) | *P* value | Peak month | Amplitude[b] (%) | *P* value |
| Sex | | | | | | | | |
| Male | 8895 | 56.2 | Mar | 2.6 | 0.22 | Mar | 2.2 | 0.35 |
| Female | 6940 | 43.8 | Jan | 2.0 | 0.49 | Dec | 2.0 | 0.52 |
| Age at diagnosis | | | | | | | | |
| < 1 year | 940 | 5.9 | Sep | 8.8 | 0.16 | Sep | 8.2 | 0.20 |
| 1–4 years | 7946 | 50.2 | Feb | 2.4 | 0.31 | Feb | 2.5 | 0.29 |
| 5–9 years | 4259 | 26.9 | Mar | 5.2 | 0.06 | Mar | 4.3 | 0.13 |
| 10–14 years | 2690 | 17.0 | Jul | 1.5 | 0.85 | Feb | 2.9 | 0.57 |
| Year of birth | | | | | | | | |
| < 1960 | 2668 | 16.8 | Feb | 7.8 | 0.02 | Feb | 8.1 | 0.01 |
| 1960–69 | 4665 | 29.5 | Jan | 0.7 | 0.95 | Feb | 0.9 | 0.92 |
| 1970–79 | 4083 | 25.8 | Jan | 2.7 | 0.49 | Dec | 2.7 | 0.48 |
| 1980–89 | 3653 | 23.1 | Jul | 3.2 | 0.40 | Aug | 3.6 | 0.30 |
| ≥ 1990 | 766 | 4.8 | Mar | 11.1 | 0.09 | Feb | 2.6 | 0.88 |
| Cell type | | | | | | | | |
| Lymphocytic | 11591 | 73.2 | Feb | 2.8 | 0.11 | Feb | 2.8 | 0.11 |
| Other | 4244 | 26.8 | May | 2.4 | 0.53 | Jul | 2.4 | 0.53 |
| Immunophenotype[c] | | | | | | | | |
| Common | 4293 | 62.8 | Dec | 1.5 | 0.80 | Feb | 2.9 | 0.42 |
| B-cell | 89 | 1.3 | Aug | 13.9 | 0.65 | Aug | 12.5 | 0.71 |
| T-cell | 735 | 10.7 | Oct | 3.7 | 0.78 | Jul | 2.9 | 0.86 |
| Null-cell | 334 | 4.9 | Sep | 19.1 | 0.05 | Sep | 17.2 | 0.08 |
| Unknown | 1387 | 20.3 | Sep | 7.1 | 0.17 | Sep | 6.4 | 0.24 |
| Total | 15835 | 100 | Feb | 1.9 | 0.25 | Feb | 1.5 | 0.43 |

[a]Adjusted for birth cohort trend in the incidence of leukaemia. [b]Amplitude = % increase in rate during peak month compared to mean rate for whole year. [c]Analyses restricted to lymphocytic leukaemias diagnosed from 1976 onwards.

lymphocytic cases diagnosed from 1976 onwards, the majority (63%) of which were common cell. Of the children diagnosed from 1971 onwards, 289 (2.6%) had Down's syndrome.

In general, the adjustment for trends over time made little difference to the month of birth results. The peaks, amplitudes and *P* values reported in the text below relate to the adjusted results. For all cases combined, the analyses showed a non-significant peak for subjects born in February (Figure 1 (A)). The amplitude for all cases combined was 1.5% (95% CI: -0.7% to 3.7%). The peak occurred in March for males and in December for females and occurred later in the year (September) with greater amplitude (8.2%) among cases diagnosed during infancy than among older children. There was a contrast in both the timing and magnitude of the peak between birth cohorts. The peak month of birth among cases born before 1960 was February, this peak being significantly raised (amplitude = 8.1%, $P = 0.01$). Further investigation of this early birth cohort showed that the significant variation in birth dates was confined to cases that were diagnosed before 1962, the period during which virtually all cases were ascertained through death certification and dates of diagnosis were obtained retrospectively. There was no evidence of seasonal variation among cases born during later birth periods. The peak in month of birth was not significant for either histological sub-grouping. When the lymphocytic cases were examined according to immunophenotype, some evidence ($P = 0.08$) was found of a September peak (amplitude = 17.2%) among null-cell cases. Further age stratification of the lymphocytic cases into 1–6 years and 7+ years showed no evidence of seasonality in the month of birth in either age group (not shown in table). There was also no evidence of seasonality when infant leukaemias were subdivided into cases diagnosed during the first 6 months of life or cases diagnosed thereafter (not shown in table).

Table 2 shows the results of the month of diagnosis analyses, unadjusted and adjusted for trends in incidence over time. In general, the adjustment tended to reduce the amplitude of the observed peaks. The unadjusted analyses found some suggestion ($P = 0.08$) of a summer peak in the month of diagnosis for all cases combined but when adjusted for time trends, this was less evident (August peak, $P = 0.13$, amplitude = 2.3% (95% CI: 0.1% to 4.5%) (Figure 1 (B)). This summer peak was apparent, although non-significant, in most of the analytical subgroups but was most evident among females ($P = 0.06$) and among cases diagnosed before 1962 ($P = 0.02$). There was no evidence of seasonality in month of birth or month of diagnosis when the above analyses were repeated on the 289 children who had Down's Syndrome (not shown in table).

When analyses were repeated on the 26 944 non-leukaemia cases there was no evidence of seasonality in either month of birth (adjusted $P = 0.34$) or month of diagnosis (adjusted $P = 0.41$) for all cases combined or for those born before 1960 (not shown in table). Since incidence data were not available on non-leukaemia cases before 1962 the analyses that produced a significant result among the leukaemia cases for this period could not be replicated for other cancers.

 © 2001 Cancer Research Campaign

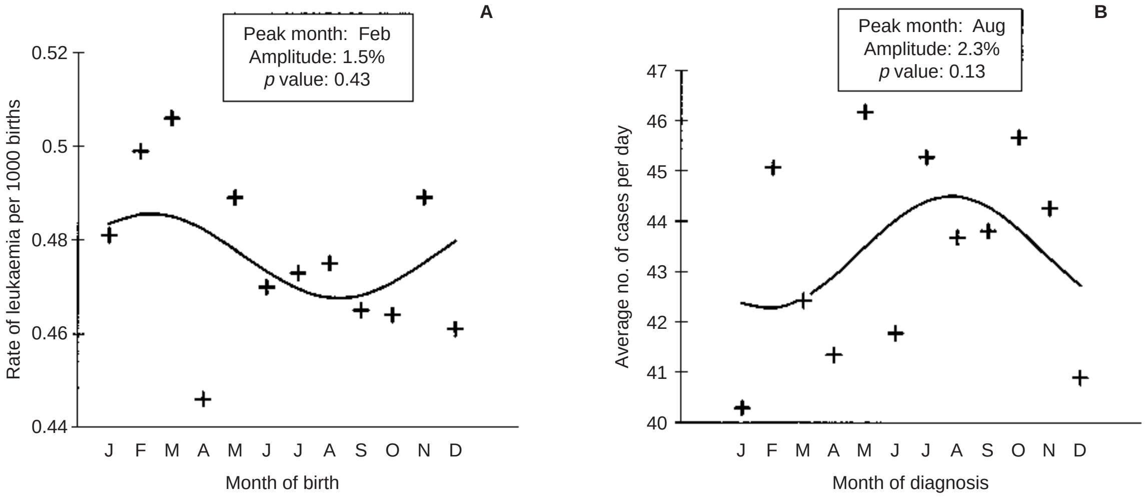

**Figure 1** Seasonal variation in (**A**) month of birth and (**B**) month of diagnosis of leukaemia cases (observed rates (+) and fitted rates from adjusted harmonic model (−)).

**Table 2** Seasonality of month of diagnosis

| | | **Harmonic analyses** | | | | | |
|---|---|---|---|---|---|---|---|
| | | **Unadjusted** | | | **Adjusted[a]** | | |
| | **No.** | **Peak month** | **Amplitude[b] (%)** | ***P* value** | **Peak month** | **Amplitude[b] (%)** | ***P* value** |
| Sex | | | | | | | |
| Male | 8895 | Aug | 1.8 | 0.50 | Sep | 1.3 | 0.67 |
| Female | 6940 | Jul | 4.1 | 0.05 | Jul | 4.0 | 0.06 |
| Age at diagnosis | | | | | | | |
| < 1 year | 940 | Aug | 2.5 | 0.86 | Aug | 2.3 | 0.88 |
| 1–4 years | 7946 | Aug | 3.5 | 0.08 | Jul | 3.3 | 0.12 |
| 5–9 years | 4259 | Jul | 4.0 | 0.18 | Jul | 3.8 | 0.22 |
| 10+ years | 2690 | Feb | 2.9 | 0.58 | Mar | 3.4 | 0.46 |
| Year of diagnosis | | | | | | | |
| < 1962 | 1282 | Aug | 10.8 | 0.02 | Aug | 10.8 | 0.02 |
| -⩾ 1962 | 14553 | Jul | 1.9 | 0.27 | Jun | 1.7 | 0.36 |
| Cell type | | | | | | | |
| Lymphocytic | 11591 | Aug | 3.0 | 0.07 | Jul | 2.5 | 0.16 |
| Other | 4244 | Jun | 2.6 | 0.48 | Jun | 2.6 | 0.49 |
| Immunophenotype[c] | | | | | | | |
| Common | 4293 | Sep | 3.3 | 0.32 | Sep | 1.9 | 0.69 |
| B-cell | 89 | May | 22.8 | 0.32 | May | 22.4 | 0.30 |
| T-cell | 735 | Jul | 5.2 | 0.61 | Jun | 4.9 | 0.64 |
| Null-cell | 334 | Jan | 5.5 | 0.78 | Jan | 6.0 | 0.74 |
| Unknown | 1387 | Jul | 4.3 | 0.53 | Jul | 4.2 | 0.54 |
| Total | 15835 | Jul | 2.6 | 0.08 | Aug | 2.3 | 0.13 |

[a]Adjusted for temporal trend in the incidence of leukaemia. [b]Amplitude = % increase in rate during peak month compared to mean rate for whole year. [c]Analyses restricted to lymphocytic leukaemias diagnosed from 1976 onwards.

### DISCUSSION

Using data from a large population-based registry we found no evidence of seasonal variation in either the birth or diagnosis dates of children overall born and diagnosed with leukaemia between 1953 and 1995. We did, however, find evidence of a February peak in the month of birth and an August peak in the month of diagnosis among cases that were born and diagnosed during the early period of the study.

Because the data are from a population-based registry that had a high level of completeness and accuracy throughout the study period, the main results are unlikely to have been influenced by

*© 2001 Cancer Research Campaign* 

ascertainment or recording bias. The latter conclusion is reinforced by the lack of seasonality when the analyses were repeated on all cancers other than leukaemia. For the month of diagnosis analyses, we took account of possible delays in registration caused by the Christmas and New Year holiday period by using the mid-point of each month as the analytic time period. We also took account of temporal changes in the number of diagnosed cases by including a quadratic term in each of the cosinor models. Previous studies have not taken account of both of these issues.

The analyses were based on over 15 000 cases of childhood leukaemia so the failure to find significant seasonal variation in the birth dates for all cases combined is not due to a lack of statistical power. Also, the small amplitude (1.5%) estimated from the harmonic curve together with a narrow confidence interval suggests that even if there is seasonal variation it is very small. Our negative finding accords with the results of far smaller previous studies that have analysed seasonality of birth dates of childhood leukaemias over a wide age range (Van Steensel-Moll, 1983; Meltzer at al, 1996; Badrinath et al, 1997). As aetiology of childhood leukaemia may well vary by histological type or age, such overall analyses leave open the possibility that seasonality might be confined to particular subgroups. When we carried out separate month of birth analyses according to age, sex and histology, however, we found no evidence of variation in any of the subgroups. The sex and histology results are in accordance with previous studies that have stratified by these factors (Bailar and Gurian, 1964; Van Steensel-Moll et al, 1983). Studies that have carried out age-specific analyses have produced contrasting results, some (e.g. Vianna and Polan, 1976) have found a different significant peak in each of the age groups examined whilst others have failed to find seasonality in any age group (Bailar and Gurian, 1964; Stark and Mantel, 1967; Van Steensel-Moll et al, 1983). Perhaps of most interest with regard to age-specific analyses is our failure to find seasonal variation in the birth dates of infant leukaemias, an age-group in which previous studies have found significant seasonality (Ederer et al, 1965; Meltzer et al, 1989). We also found no evidence to support Stewart's hypothesis (1975) that a deficit of leukaemia cases may occur among babies born between July and December who are exposed to winter conditions during the first 6 months of life and are therefore more likely to die from respiratory infections before a diagnosis of leukaemia is made.

We did find significant variation in the birth dates of cases born during the early period of the study, a result that remained significant after adjustment for temporal trends. Various possible explanations can be postulated. First, the result could be due to chance given the number of statistical tests we have carried out. Second, it could be due to Stewart's hypothesis that seasonal variation has disappeared because of the changing seasonality in the occurrence of, or fatality from, a common winter infection such as pneumonia. Third, it might be due to a causative agent acting during gestation or very shortly after birth becoming less seasonal over time. The most likely seasonal risk factors are common infections and allergies. Seasonality data exist over time for a number of common infections (e.g. influenza, varicella) and allergies (e.g. pollen) but none, as far as we know, have undergone secular changes in seasonality of a nature to support this hypothesis. Finally, there is a growing body of evidence that the mixing of individuals from different urban–rural origins or different socio-economic backgrounds may facilitate the transmission of a, yet unknown, infectious agent leading to increased leukaemia rates (Kinlen, 1995; Dickinson and Parker, 1999). If this hypothesis is correct and if immunity has become more widespread over time as populations have become more 'mixed' then this could explain why seasonality was found only during the early period. Alternatively, Greaves (1997) has suggested that leukaemia may arise as a consequence of an abnormal immunological response to late infection. Thus, increased population mixing and/or changes in immunological response to late infections may explain the disappearance of seasonal variation in childhood leukaemia. Population-mixing effects have generally been observed in rural communities into which a large number of urban dwellers have migrated. Analyses that categorized cases into urban or rural status would therefore have been interesting but the difficulty in obtaining consistent definitions over such a long study period precluded such analyses in the present study.

A large number of studies have investigated seasonal variation in the onset or diagnosis dates of childhood leukaemia, with conflicting results. The underlying rationale behind these studies has been that if a seasonal causative agent is involved in the aetiology of childhood leukaemia and its induction period is sufficiently short, then a similar seasonal pattern should be observed in the leukaemia incidence rates. Seasonality could also occur if diagnosis is more rapid at some times of the year than others. The present study found evidence of seasonal variation in the diagnosis dates of cases diagnosed during the early period of the study but not during later periods. As with the season of birth analyses, the amplitude for all cases combined was low (2.3%) with a narrow confidence interval and so the number of cases that can potentially be attributable to the seasonal variation would therefore be very few.

Findings in previous studies, all of which have been much smaller than the present one (less than 50% in size) have been mixed. Several have found no seasonality (Bjelke, 1964; Mainwaring, 1996; Till, et al 1967; Gunz and Spears, 1968; Walker and Van Noord, 1982; Gilman et al, 1998; Thorne et al, 1998; Douglas et al, 1999) while others have found a winter peak (Hayes, 1961; Fraumeni, 1963; Lanzkoswky, 1964), summer peak (Lee, 1962; Knox, 1964; Fekety and Carey, 1969; Badrinath et al, 1997; Gilman et al, 1998, Westerbeek et al, 1998; Ross et al, 1999) or a more complex pattern (Harris and Al-Rashid, 1984; Harris et al, 1987). Most of the cases analysed by Lee (1962), and all of the cases in the other UK studies (Knox, 1964; Mainwaring, 1996; Till et al, 1967; Badrinath et al, 1997; Thorne et al, 1998; Gilman et al, 1998; Douglas et al, 1999) have been included in the present analyses, and are therefore effectively small subsets (less than 20% in size) of the present data. Some of the contrasting results may be due to the different statistical approaches that have been used, the failure to take account of temporal changes, but in particular the arbitrary cut-points that have been used to define the different seasons of the year. Reanalysing the monthly counts of cases presented in the previous UK studies we have fitted cosinor models (without adjustment for temporal trends) and found that only the study by Lee (1962) produced a significant (summer peak, $P = 0.003$) result. This study by Lee was based on cases collected through the National Cancer Registration Scheme for England and Wales, between 1946 and 1959, and there is therefore much overlap with the early period of our present analyses in which we also found a summer peak. When we carried out analyses on our own data, using the same summer: winter ratio approach and seasonal cut-points employed by these previous studies, we also found a significant ($P < 0.001$) excess of cases diagnosed during

 © 2001 Cancer Research Campaign the summer months. These findings illustrate the sensitivity of the results to the chosen method of analysis, the choice of arbitrary cut-points and whether account is taken of temporal changes. Interestingly, the one UK study (Westerbeek et al, 1998) that employed the same analytical technique as ourselves found the same borderline significant result that we observed before we adjusted for temporal trends.

The finding of seasonal variation in diagnosis dates during the early period of the study should be interpreted with some caution since these cases were ascertained through death certificates and dates of diagnoses obtained retrospectively from hospital records. It is possible that apparent seasonality in these cases arose because of a complication of death (Stewart and Kneale, 1969) but alternatively the finding could be due, as before, to the effects of population mixing over time and/or changes in the seasonality of exposure, or immunological response, to an infectious agent.

A potential limitation of this study is that the absence of significant seasonal variation in the diagnosis dates for all cases combined could be due to the fact that we examined seasonality in the date of diagnosis rather than the date of clinical onset. Westerbeek et al (1998) found evidence of seasonality in the date of first symptom for cases of common-ALL but no evidence of seasonality in the date of diagnosis. Even modest seasonal variation in the lag period between first symptom and clinical diagnosis could explain why date of diagnosis could be an inappropriate temporal measurement when analysing seasonality of leukaemia.

In summary, this study found no overall seasonal pattern in either the birth or incidence dates of childhood leukaemia. Significant seasonal variation was found among cases born and diagnosed during the early period of the study, a result that may indicate temporal changes in the seasonality of exposure, or immunological response, to an infectious agent.

### ACKNOWLEDGEMENTS

We would like to thank Dr GJ Draper for his valuable advice on the study design and analysis. We are also very grateful to Mr TJ Vincent for producing the data files for analysis and making them so readily available. Our thanks also go to Mr M Shipley and Mr C Frost for their useful statistical advice and to Mr Z Qiao for his valuable contribution to the computing aspects of this project. Mr C Higgins is funded by a Medical Research Council programme grant. The NRCT receives notifications of leukaemia cases from regional and national cancer registries, regional children's tumour registries, the UK Children's Cancer Study Group, the Clinical Trial Service Unit, the Office for National Statistics and the Registrar General for Scotland. The CCRG is supported by the Departments of Health for England and Wales and for Scotland. Views expressed in this publication are not necessarily those of the Department of Health.

### REFERENCES

Badrinath P, Day NE and Stockton D (1997) Seasonality in the diagnosis of acute lymphocytic leukaemia. *Br J Cancer* **75**: 1711–1713

Bailar JC and Gurian JM (1964) Month of birth and cancer mortality. *J Natl Cancer Inst* **33**: 237–242

Bjelke E (1964) Leukaemia in children and young adults in Norway. *Cancer* **17**: 248–255

Dickinson HO and Parker L (1999) Quantifying the effect of population mixing on childhood leukaemia risk: the Seascale cluster. *Br J Cancer* **81**: 144–151

Doll R (1999) The Seascale cluster: a probable explanation. *Br J Cancer* **81**: 3–5

Douglas S, Cortina-Borja M and Cartwright R (1999) A quest for seasonality in presentation of leukaemia and non-Hodgkin's lymphoma. *Leuk Lymph* **32**: 523–532

Ederer F, Miller RW, Scotto J and Bailar JC (1965) Birth-month and infant cancer mortality (letter to the editor). *Lancet* **2**: 185–186

Edwards JH (1961) The recognition and estimation of cyclic trends. *Ann Hum Genet Lond* **25**: 83–87

Fekety FR and Carey JJH (1969) Season and the onset of acute childhood leukaemia. *Maryland State Med J* **11**: 73–77

Fraumeni JF (1963) Seasonal variation in leukaemia incidence (letter to the editor). *Br Med J* **2**: 1408–1409

Gilman EA, Sorahan T, Lancashire RJ, Lawrence GM and Cheng KK (1998) Seasonality in the presentation of acute lymphoid leukaemia (letter to the editor). *Br J Cancer* **77**: 677–678

Greaves MF (1997) Aetiology of acute leukaemia. *Lancet* **349**: 344–349

Gunz and Spears (1968) Distribution of acute leukaemia in time and space. Studies in New Zealand. *Br Med J* **4**: 604–608

Harris RE and Al-Rashid R (1984) Seasonal variation in the incidence of childhood acute lymphocytic leukaemia in Nebraska. *Nebraska Med J* **6**: 192–198

Harris RE, Harrell FE, Kashineth DP and Al-Rashid R (1987) The seasonal risk of pediatric/ juvenile acute lymphocytic leukemia in the United States. *J Chron Dis* **40**: 915–923

Hayes DM (1961) The seasonal incidence of acute leukaemia. *Cancer* **14**: 1301–1305

Kellett CE (1937) Acute myeloid leukaemia in one of identical twins. *Arch Dis Childh* **12**: 239–252

Kinlen LJ (1995) Epidemiological evidence for an infective basis in childhood leukaemia. *Br J Cancer* **71**: 1–5

Knox G (1964) Epidemiology of childhood leukaemia in Northumberland and Durham. *Brit J Prev Soc Med* **18**: 17–24

Lanzkowsky P (1964) Variation in leukaemia incidence (letter to the editor). *Br Med J* **1**: 910

Lee JAH (1962) Seasonal variation in clinical onset of leukaemia in young people. *Br Med J* **1**: 1737–1738

Machin D and Gao F (2000) Seasonal variations in the diagnosis of childhood cancer (letter to the editor). *Br J Cancer* **83**: 699–700

Mainwaring D (1996) Epidemiology of acute leukaemia of childhood in the Liverpool area. *Brit J Prev Soc Med* **20**: 189–194

Marrero O (1983) The performance of several statistical tests for seasonality in monthly data. *J Statist Comput Simul* **17**: 275–296

Meltzer AA, Spitz MR, Johnson CC and Culbert SJ (1989) Season-of-birth and acute leukaemia of infancy. *Chronobiol Int* **6**: 285–289

Meltzer AA, Annegers JF and Spitz MR (1996) Month of birth and incidence of acute lymphoblastic leukaemia in children. *Leuk Lymph* **23**: 85–92

Office of Population Censuses and Surveys (1976–1997). *Birth Statistics 1974–1995*. Series FM1 no. 1–24. HMSO, London

Registrar General (1955–1959) *Registrar General's Statistical Review of England and Wales, 1953–57*. Tables. Part II. Civil. HMSO, London

Registrar General (1960–1975) *Registrar General's Statistical Review of England and Wales, 1958–73*. Tables. Part II. Tables, Population. HMSO, London

Ross JA, Severson RK, Swensen AR, Pollock BH, Gurney JG and Robison LL (1999) Seasonal variations in the diagnosis of childhood cancer in the United States. *Br J Cancer* **81**: 549–553

Stark CR and Mantel N (1967) Temporal-spatial distribution of birth dates for Michigan children with leukaemia. *Cancer Res* **27**: 1749–1755

StataCorp (1997) *Stata Statistical Software: Release 5.0*. College Station, TX: Stata Corporation

Stewart A (1975) Infant leukaemias and cot deaths. *Br Med J* **2**: 605–607

Stewart A and Kneale GW (1969) Role of local infections in the recognition of haemopoietic neoplasms. *Nature* **223**: 741–742

Stiller CA, Allen MB and Eatock EM (1995) Childhood cancer in Britain: the National Registry of Childhood Tumours and incidence rates 1978–1987. *Eur J Cancer* **31**: 2028–2034

Thorne R, Hunt LP and Mott MG (1998) Seasonality in the diagnosis of childhood acute lymphoblastic leukaemia (letter to the editor). *Br J Cancer* **77**: 678

Till MM, Hardisty RM, Pike MC and Doll R (1967) Childhood leukaemia in Greater London: a search for evidence of clustering. *Br Med J* **3**: 755–758

Van Steensel-Moll HA, Valkenburg HA, Vandenbroucke JP and Van Zanen GE (1983) Time space distribution of childhood leukaemia in the Netherlands. *J Epidemiol Comm Health* **37**: 145–148

Vianna NJ and Polan AK (1976) Childhood lymphatic leukaemia: Prenatal seasonality and possible association with congenital varicella. *Am J Epidemiol* **103**: 321–332

*© 2001 Cancer Research Campaign* 

Walker AM and Van Noord PAH (1982) No seasonality in the diagnosis of acute leukeamia in the United States. *J Natl Cancer Inst* **69**: 1283–1287

Westerbeek RMC, Blair V, Eden OB, Stevens RF, Will AM, Taylor GM and Birch JM (1998) Seasonal variations in the onset of childhood leukaemia and lymphoma. *Br J Cancer* **78**: 119–124

*© 2001 Cancer Research Campaign*
